# Requests for Medical Assistance in Dying by Young Dutch People With Psychiatric Disorders

**DOI:** 10.1001/jamapsychiatry.2024.4006

**Published:** 2025-01-02

**Authors:** Lizanne J.S. Schweren, Sanne P.A. Rasing, Monique Kammeraat, Leah A. Middelkoop, Ruthie Werner, Saskia Y.M. Mérelle, Julian M. Garcia, Daan H.M. Creemers, Sisco M.P. van Veen

**Affiliations:** 1 113 Suicide Prevention, Amsterdam, the Netherlands; 2 Center for Youth Depression, GGZ Oost Brabant, Boekel, the Netherlands; 3 Behavioural Science Institute, Radboud University, Nijmegen, the Netherlands; 4 Expertisecentrum Euthanasie, The Hague, the Netherlands; 5 Department of Psychiatry, Spaarne Gasthuis, Haarlem, the Netherlands; 6 Department of Psychiatry, Amsterdam University Medical Centre, Amsterdam, the Netherlands

## Abstract

**Importance:**

In the Netherlands, a growing group of young people request medical assistance in dying based on psychiatric suffering (MAID-PS). Little is known about this group, their characteristics, and outcomes.

**Objective:**

To assess the proportion of requests for and deaths by MAID-PS among young patients, outcomes of their application and assessment procedures, and characteristics of those patients who died by either MAID or suicide.

**Design, Setting, and Participants:**

This retrospective cohort study included Dutch individuals younger than 24 years requesting MAID-PS between January 1, 2012, and June 30, 2021, whose patient file had been closed by December 1, 2022, at the Expertisecentrum Euthanasie, a specialized health care facility providing MAID consultation and care.

**Main Outcomes and Measures:**

Outcomes of the MAID-PS assessment procedure (discontinued, rejected, or MAID-PS) and clinical characteristics of patients who died by MAID or suicide.

**Results:**

The study included 397 processed applications submitted by 353 individuals (73.4% female; mean [SD] age, 20.84 [1.90] years). Between 2012 and the first half of 2021, the number of MAID-PS applications by young patients increased from 10 to 39. The most likely outcome was application retracted by the patient (188 [47.3%]) followed by application rejected (178 [44.8%]). For 12 applications (3.0%), patients died by MAID. Seventeen applications (4.3%) were stopped because the patient died by suicide during the application process and 2 (0.5%) because the patient died after they voluntarily stopped eating and drinking. All patients who died by suicide or MAID (n = 29) had multiple psychiatric diagnoses (most frequently major depression, autism spectrum disorder, personality disorders, eating disorder, and/or trauma-related disorder) and extensive treatment histories. Twenty-eight of these patients (96.5%) had a history of suicidality that included multiple suicide attempts prior to the MAID application. Among 17 patients who died by suicide, 13 of 14 (92.9%) had a history of crisis-related hospital admission, and 9 of 12 patients who died by MAID (75.0%) had a history of self-harm.

**Conclusions and Relevance:**

This cohort study found that the number of young psychiatric patients in the Netherlands who requested MAID-PS increased between 2012 and 2021 and that applications were retracted or rejected for most. Those who died by MAID or suicide were mostly female and had long treatment histories and prominent suicidality. These findings suggest that there is an urgent need for more knowledge about persistent death wishes and effective suicide prevention strategies for this high-risk group.

## Key Points

**Question:**

What are outcomes in young people requesting medical assistance in dying based on psychiatric suffering (MAID-PS)?

**Findings:**

In this cohort study of 397 applications for MAID-PS by 353 Dutch people younger than 24 years between 2012 and 2021, 47% of applications were retracted and 45% were rejected. For 3% of applications, patients died by MAID, and for 4%, the patient died by suicide during the application process.

**Meaning:**

The findings suggest that there is an urgent need for more knowledge about persistent death wishes and effective suicide prevention strategies for this group.

## Teaser

This cohort study examines outcomes of requests for medical assistance in dying among Dutch young people with psychiatric disorders.

## Introduction

Dutch law provides a comprehensive framework to, under specific circumstances, allow medical assistance in dying (MAID) for patients with unbearable and irremediable suffering, including patients with psychiatric disorders.^1^ Dutch law exempts physicians from prosecution who terminate the life of a patient or aid a patient in terminating their own life if the physician is convinced that the patient’s request is voluntary and well considered and the patient’s suffering is unbearable, with no prospect of improvement, and they have informed the patient about their situation and prognosis; come to the conclusion, together with the patient, that there is no reasonable alternative in the patient’s situation; consulted at least one other independent physician, who must see the patient and give a written opinion on whether the aforementioned criteria have been fulfilled; and exercised due medical care and attention in terminating the patient’s life or assisting in the patient’s suicide.

The number of people dying by MAID based on psychiatric suffering (MAID-PS) in the Netherlands increased from 41 in 2014 (0.2 per 100 000 inhabitants) to 138 in 2023 (0.8 per 100 000 inhabitants).^2^ At the same time, the proportion of deaths by MAID-PS among all deaths by MAID remained low (0.8% in 2014; 1.5% in 2023). Yearly suicide rates in the Netherlands have, since 2014, been relatively stable, with an average of 10.8 per 100 000 inhabitants (range, 10.4-11.2 per 100 000 inhabitants).^3^ Patients requesting MAID-PS are often female, have multiple psychiatric diagnoses, and have a long history of psychiatric treatment.^4^

Among those requesting MAID-PS are a substantial number of young persons. Dutch law grants persons aged 16 years or older the right to independent medical decision-making and children aged 12 to 16 years the right to shared decision-making alongside their parents or guardians. For multiple reasons, MAID-PS assessment in young persons is complicated. For one, assessment of irremediability of the suffering is difficult in patients undergoing developmental and social changes.^5^ Also, irremediability of mental illness is controversial, especially when a young and physically healthy patient might be expected to live for many years, during which new treatments might be discovered.^6^ In addition, ongoing neurobiological development during adolescence may affect competency of decision-making.^7^

Little has been written about young people requesting MAID-PS. Persistent death wishes in adolescents are typically interpreted as persistent or chronic suicidality, which is associated with increased hopelessness,^8^ emotion regulation problems,^9^ early-onset self-harm,^10^ and co-occurring psychiatric diagnoses.^11^ However, suicidality alone is an insufficient reason for MAID-PS.^12^ Moreover, suicidality might complicate MAID assessment: the fluctuating nature of suicidality^13^ may hamper assessment of the consistency of the death wish,^14^ and acute suicidality might interfere with decision-making.^15^

As individuals, medical professionals, and societies worldwide struggle with ethical questions regarding MAID-PS, especially in young persons, there is an urgent need for more knowledge about this group. We assessed the proportion of requests for and deaths by MAID-PS among young patients (aged <24 years) in the Netherlands, outcomes of application and assessment procedures in this group, and characteristics of those patients who died by either MAID-PS or suicide.

## Methods

### Data Source and Study Population

The current retrospective cohort study was based on patient files from Expertisecentrum Euthanasie, the Dutch national expertise center for euthanasia. Expertisecentrum Euthanasie provides MAID consultation and care for patients who cannot receive these services from their own physician. It constitutes insured care, meaning all residents of the Netherlands have access. Between 2013 and 2021, an estimated 64% of all MAID-PS cases in the Netherlands were handled by Expertisecentrum Euthanasie.^16^ This percentage is likely higher for relatively complex cases, such as young patients. Patients may reapply with Expertisecentrum Euthanasie after rejection. This study was exempt from patient informed consent because it concerned review of deidentified patient data only, as was confirmed by the ethical board of the Amsterdam University Medical Center. This report followed Strengthening the Reporting of Observational Studies in Epidemiology (STROBE) reporting guideline.^17^

Applications meeting the following criteria were included: (1) the applicant requested MAID based primarily on psychiatric suffering; (2) the applicant was younger than 24 years at the time of application; (3) the application occurred between January 1, 2012, and June 30, 2021; and (4) the application had been closed by December 1, 2022 (ie, data extraction date). The age of 24 years was chosen as a cutoff as it approximates both a change in neurodevelopmental processes (eg, maturation of the prefrontal cortex) and the transition to adult mental health care services in the Netherlands. Cases were deemed closed when (1) the application was discontinued by the patient, (2) the application was rejected by Expertisecentrum Euthanasie, or (3) the patient died.

### Application Process

MAID-PS applications go through several steps to evaluate the patients’ eligibility for MAID. Each step results in a number of patients either halting their application (for any reason, including death by suicide or natural causes) or being rejected and a number of patients proceeding to the next step. Although over the years, administrative procedures have changed in response to increasing demands, the steps can generally be described as follows.

The initial request consists of the patient (or, at the patients request, their physician or relative) completing an application form detailing, among other items, their illness history and reason(s) for applying. Next, the patient and their physician(s) are requested to provide full medical files, resulting in a complete application. Next, the application proceeds to an in-person eligibility screening by a psychiatrist. Applications that could potentially meet eligibility criteria proceed to the full assessment phase, for which there is usually a waiting period. To ascertain irremediability of the suffering, patients may be requested to undergo treatment during the assessment phase. Full assessment may take up to several years to complete and results in an eligibility decision and, if granted, MAID.

### Exposures, Outcomes, and Covariates

For each application, we extracted year and month of application and sex (male, female). For each patient, we registered their total number of applications within the study period. The outcome of each MAID application procedure was determined at file closure as (1) patient discontinued due to incomplete medical file, (2) patient discontinued before eligibility screening, (3) patient discontinued during or after eligibility screening but before full assessment, (4) patient discontinued during or after full assessment, (5) patient discontinued at unknown time, (6) application rejected after eligibility screening, (7) application rejected after full assessment, (8) application rejected at unknown time, or (9) MAID. Outcomes 1 through 5 were grouped to constitute a group of all applications that were discontinued by the patient. Outcomes 6 through 8 were grouped to constitute all applications that were rejected. Stringent privacy regulations did not allow extraction of more details (eg, psychiatric diagnoses, reason for rejection).

Suicide was reported when the patient died by suicide during the MAID application or assessment procedure. Voluntarily stopping eating and drinking (VSTED) during the procedure was registered separately, as Dutch regulations qualify VSTED as death by natural causes. The small number of VSTED cases (n = 2) did not allow separate reporting of clinical characteristics in this group without potential breach of patient privacy. Deaths by suicide or VSTED occurring after rejection or discontinuation were not reliably documented in the (then closed) patient file; thus, these were not investigated.

For applicants who had died by MAID or suicide, we attempted to obtain detailed information regarding their history of mental health problems, including suicidality and their treatment history, from the patient files. Access to sensitive patient data regarding these themes (but not regarding other themes) was considered ethically permissible, as they could contribute to answering questions with direct and urgent clinical implications. The following clinical characteristic variables were sought: psychiatric diagnoses that applied at any time before or during the MAID application procedure, childhood sexual abuse (yes or no), childhood bullying (yes or no), history of self-harm (yes or no), self-reported number of suicide attempts prior to MAID request, and age at first suicide attempt. For treatment history, we sought to assess whether the following interventions had been tried (yes or no): selective serotonin reuptake inhibitors, monoamine oxidase inhibitors, tricyclic antidepressants, lithium, antipsychotic medications, sedatives or sleep medications, electroconvulsive therapy, deep brain stimulation, intensive home treatment, eye movement desensitization and reprocessing therapy, and recovery-oriented care (eg, psychosocial support, rehabilitative care, or assisted living). We further assessed the number of unique psychoactive pharmacological interventions tried, number of unique antidepressants tried, history of crisis-related hospital admission (yes or no), history of inpatient treatment (not crisis related; yes or no), history of involuntary treatment (eg, care authorization, compulsory medication, physical restraint, and coercive feeding; yes or no), and age at first contact with any mental health professional.

Information was retrieved from unstructured documentation (eg, referral letters, meeting notes), leading to missing data. In addition, most patients who died by suicide had not undergone full assessment at the time of their death, resulting in a substantial amount of missing data in this group. We reported results for all variables with 70% or greater valid data per group (MAID, suicide).

### Statistical Analysis

We provide descriptive characteristics of all included MAID-PS applications and patients submitting these applications as well as the distribution of application outcomes. Furthermore, we describe clinical and treatment characteristics of patients who died by either MAID or suicide. Denominators may vary owing to missing data. Data were analyzed using RStudio, version R4.4.1 (RStudio).

## Results

### Sample Characteristics

**Figure 1.**
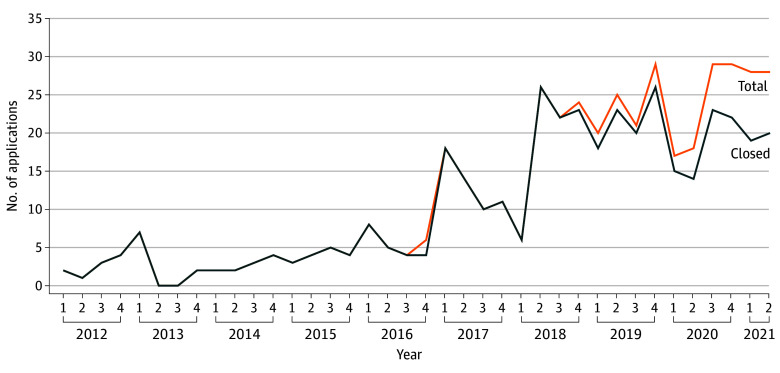
Medical Assistance in Dying Based on Psychiatric Suffering Applications by Year and Quarter of Application

Of all 444 MAID-PS applications filed within the inclusion period, 397 (89.4%) had been closed at the time of data extraction. A total of 397 applications by 353 unique patients met the inclusion criteria. The majority of patients were female (259 [73.4%]); 94 (26.6%) were male. Mean (SD) age was 20.84 (1.90) years. Most patients submitted 1 application (314 [88.9%]) during the study period, while few patients submitted 2 (34 [9.6%]) or 3 (5 [1.4%]) applications. Stratification by quarter and year (Figure 1) showed an increase with time from 10 applications in 2012 to 74 applications in 2020 and 39 applications in the first half of 2021. Note that in later years, more applications were excluded from the current study as they had not yet been closed at time of data extraction.

### Outcomes of the Procedure

**Figure 2.**
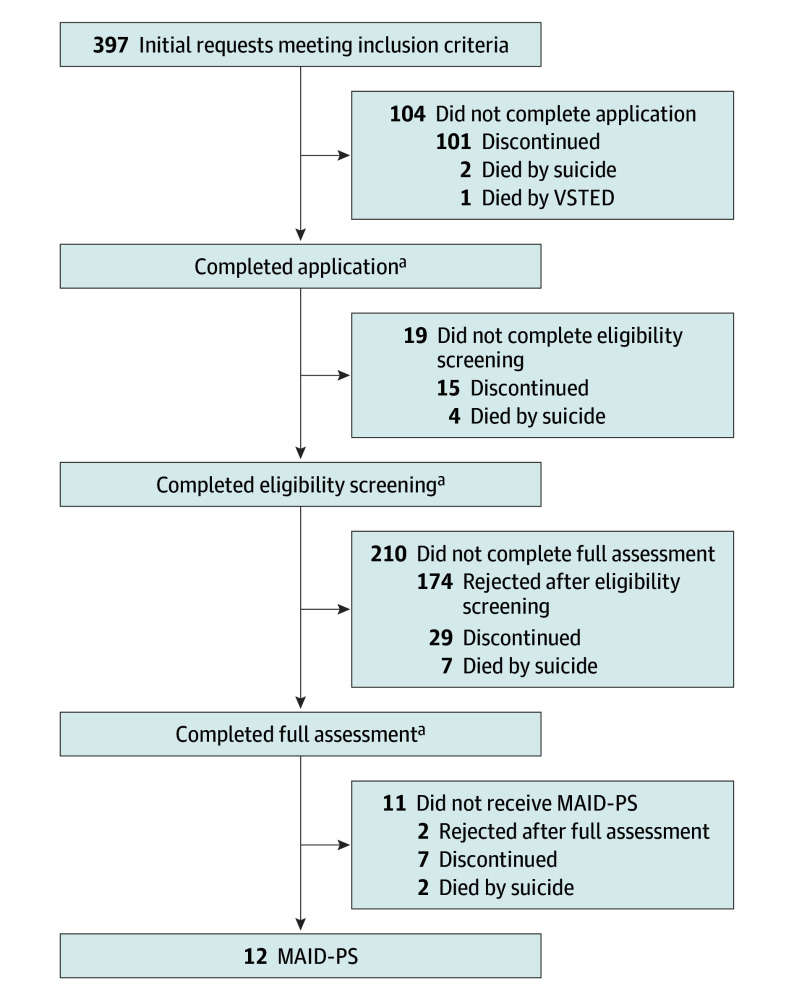
Outcomes of the Medical Assistance in Dying (MAID) Procedure Among 397 Closed Cases MAID-PS, MAID based on psychiatric suffering; VSTED, voluntarily stopping eating and drinking. ^a^Total number unavailable as 41 applications had an outcome at an unknown time: 36 discontinued, 2 died by suicide, 2 rejected, and 1 died by VSTED.

In total, 188 applications (47.3%) were discontinued by the patient (Figure 2). Most of those applications (101 [53.7%]) were halted before the initial application had been completed; 174 (60.0%) were rejected after eligibility screening. Seventeen applications (4.3%; 13 [3.3%] for females) stopped because the applicant died by suicide, and 2 (0.5%) stopped because the patient died by VSTED. Suicide occurred in all stages of the application process (mean [SD] time between application and suicide, 24.6 [24.1] months). Twelve applications (3.0%; 10 [2.5%] for females) resulted in MAID. Time between initial application and MAID varied between 2.3 and 90.0-months (median, 41.4 months; mean [SD], 46.1 [24.8] months).

### MAID

Patients who died by MAID (n = 12) presented with a large number of psychiatric diagnoses (median, 4 [range, 2-8]). The most frequently reported diagnoses were autism spectrum disorder, major depression, and eating disorder. Other common diagnoses were trauma-related disorder, borderline personality disorder, and other or unspecified personality disorders (Table). Six of 11 patients had experienced bullying (54.5%), and 3 of 11 had experienced sexual abuse (27.3%). All patients who died by MAID had a history of suicidality. Eleven (91.7%) had attempted suicide prior to their MAID application, with a median of 7 suicide attempts per patient (range, 3-100; n = 9) and a median age at first suicide attempt of 14.5 years (range, 11-20 years; n = 10). Nine of 12 patients (75.0%) had a history of self-harm.

**Table.** Characteristics of MAID-PS Applicants Who Died by MAID-PS or Suicide

Characteristic	Patients, No. (%)	
	MAID-PS (n = 12)	Suicide (n = 17)
Psychiatric diagnoses^a^		
Major depression	8 (66.7)	12 (75.0)
Autism spectrum disorder	9 (75.0)	6 (37.5)
Borderline personality disorder	5 (41.7)	7 (43.7)
Other or unspecified personality disorder	5 (41.7)	8 (50.0)
Eating disorder	6 (50.0)	6 (37.5)
Trauma-related disorder	5 (41.7)	7 (43.7)
Substance use or addictive disorder	2 (16.7)	1 (6.3)
Obsessive-compulsive disorder	4 (33.3)	3 (18.7)
Bipolar disorder	0	2 (12.5)
Anxiety disorder	3 (25.0)	2 (12.5)
Attention-deficit/hyperactivity disorder	4 (33.3)	2 (12.5)
Psychotic disorder	0	1 (6.3)
Dissociative disorder	1 (8.3)	1 (6.3)
Other^b^	3 (25.0)	2 (12.5)
Pharmacological treatment history^c^		
SSRI	11 (91.7)	13 (92.9)
MAO inhibitors	4 (33.3)	1 (7.1)
Tricyclic antidepressants	9 (75.0)	3 (21.4)
Lithium	7 (58.3)	2 (14.3)
Antipsychotics	11 (91.7)	10 (71.4)
Sedative or sleep medication	11 (91.7)	12 (85.7)
Neuromodulatory treatment history		
Electroconvulsive therapy	4 (33.3)	NA
Deep brain stimulation	1 (8.3)	NA

Abbreviations: MAID, medical assistance in dying; MAO, monoamine oxidase; NA, not available; SSRI, selective serotonin reuptake inhibitor.

^a^ Data were missing for 1 patient who died by suicide.

^b^ Other diagnoses included conversion disorder, impulse control disorder, and unspecified learning disorder.

^c^ Data were missing for 3 patients who died by suicide.

All patients had tried multiple pharmacological and psychotherapeutic interventions. The median age at first mental health treatment was 9 years (range, 4-18 years; n = 11). Patients had been prescribed a median of 11.5 unique psychopharmacological interventions (range, 4-19), including a median of 4 unique antidepressants (range, 2-8) (Table). Five patients (41.7%) had undergone neuromodulatory treatment (ie, electroconvulsive therapy or deep brain stimulation). Eleven patients (91.7%) had been treated in an inpatient setting, 8 (66.7%) had received intensive home treatment, 10 (83.3%) had undergone eye movement desensitization and reprocessing therapy, and 10 (83.3%) had received recovery-oriented care. All patients had experienced involuntary measures during their treatment, and 10 (83.3%) had a history of crisis-related hospital admission(s).

### Suicide

Psychiatric diagnoses were available for 16 of the 17 patients (94.1%) who died by suicide, with a median of 3 diagnoses per patient (range, 1-8; n = 16). The most frequently reported diagnosis was major depression. Other common diagnoses were borderline personality disorder, other or unspecified personality disorder, trauma-related disorder, autism spectrum disorder, and eating disorder. Fifteen of 16 patients (93.7%) had attempted suicide prior to their MAID application, and 13 of 14 (92.9%) had a history of self-harm. Eight of 12 (66.7%) had a history of sexual abuse. Almost all (13 of 14 [92.9%]) had a history of crisis-related hospital admission(s). Patients who died by suicide had been prescribed a median of 7.5 unique psychoactive medications (range, 3-11; n = 14), including a median of 1.5 unique antidepressants (range, 0-6; n = 14). The median age at first mental health treatment was 11 years (range, 6-18 years; n = 13). Due to missing data in the group that died by suicide, we could not reliably assess any other clinical or treatment-related variables.

## Discussion

We identified 397 MAID-PS applications by 353 unique patients younger than 24 years, of whom 74.3% were female. Twelve applications (3.0%) resulted in MAID. For 17 applications (4.3%), patients died by suicide, and for 2 (0.5%), patients died by VSTED during the application and assessment procedure. The remainder of applications were either halted by the patient (47.3%) or rejected (44.8%), often early in the procedure. All patients who died by suicide or MAID had multiple psychiatric diagnoses and extensive treatment histories. All but one had a history of suicidality that included multiple suicide attempts prior to the MAID application. Crisis-related hospital admissions and self-harm were common.

Between 2012 and 2021, the number of MAID-PS applications by young persons increased. Growing public acceptability of MAID might have contributed to the rising number of applications, although public attitudes toward MAID-PS specifically have been relatively stable since 2016.^18^ Alternatively, the increasing number of MAID-PS applications might reflect a deterioration of mental health among young people, especially females, in the Netherlands, as is also reflected by increasing numbers of young people with mental health conditions^19,20^ and increasing suicide rates.^3^ Furthermore, one may speculate that the growing number of MAID-PS applications might indicate growing despair among young psychiatric patients who feel their needs remain unmet, for instance when treatment options are inaccessible, inacceptable, or ineffective. Our group previously described how psychiatric treatment of girls and young women can be severely debilitated by recurrent suicidal behaviors, leading to demoralization among patients, parents, and mental health care professionals.^21^ More knowledge about unmet needs of and effective interventions for this group is required.

Only 3.0% of all applications by young people resulted in MAID, which is lower compared with the acceptance rate for MAID-PS among adults in the Netherlands.^2^ One in 4 applications was halted by the patient even before medical files had been assembled. Reasons for halting the application require further investigation. Patients might benefit from changing social circumstances or starting or restarting treatment. Other patients might not be able to comply with the necessary paperwork, including paperwork for which they depend on their medical professional. Of submitted completed medical files (n = 295) (Figure 2), 174 (60.0%) were rejected due to eligibility screening. A prior study in the Netherlands showed that the most common reason for rejection of a MAID-PS request was access to reasonable alternatives to MAID (57%), followed by the suffering not being regarded as both unbearable and irremediable (32%).^22^ Preliminary findings suggest that death wishes and poor well-being often persist after MAID requests are declined.^23^ Qualitative evidence suggests that suicidal ideation may recur when physicians fail to adequately respond to a MAID-PS request.^24^ Long-term observational studies are needed to evaluate trajectories and outcomes in young patients who either halt their application or are rejected.

We found that the young patients who died by MAID or suicide had long histories of mental health problems. Characteristics of the group that died by MAID coincide largely with those described previously in adult samples, including female sex, psychiatric multimorbidity, and the predominance of major depressive disorder, autism spectrum disorders, and personality disorders.^22,25,26,27,28,29^ Detailed descriptions of treatment histories of patients who died by MAID, by contrast, are scarce. Despite the patients’ young age, we found extensive histories of many psychological and pharmacotherapeutic treatments, including intensive second- and third-line treatments, such as electroconvulsive therapy, lithium, and inpatient psychotherapy. Despite those treatment efforts, patients experienced unbearable psychological suffering. We note that the nature of the data does not allow conclusions as to whether the patients received optimal care prior to their MAID application. Treatments might have been discontinued prematurely or poorly delivered. Moreover, more is not always better, as patients may experience negative effects of overburden and treatment fatigue.^30^ Qualitative studies are needed to further examine the role of treatment history in MAID-PS requests.

Another important finding is the prominent role of suicidality; 4.3% of patients entering the MAID-PS assessment procedure died by suicide before a decision on MAID was reached. All patients who were granted MAID had a history of suicidality, and 91.7% had a history of multiple suicide attempts. These percentages are remarkably high compared with prior studies among adults.^25^ While acute suicidal behaviors typically fluctuate,^31,32^ patients need to prove a well-considered and recurrent death wish to be eligible for MAID. In a recent qualitative report, patients with persistent death wishes described those as stable and well-considered, while they described their suicidality as a loss of control related to entrapment.^33^ Our findings urge further investigation into different death wishes that might be expressed by young people, their longitudinal trajectories, and thereby, their prospects of recovery. Our findings also emphasize the need for suicide prevention among young patients requesting MAID-PS, which we argue based on the current findings, should be perceived as an ultra high–risk group for suicide.

### Limitations

Several limitations should be considered. Importantly, detailed clinical information was only available for patients who died by suicide or MAID. Adhering to stringent privacy regulations, we could not access relevant information regarding applications of people who were not known to be dead, including (but not limited to) exact age, psychiatric diagnoses, and reason for rejection or withdrawal of the application. It is to be determined whether the clinical characterization described herein applies to the full group of applicants or whether this clinical presentation is specifically prevalent in those at high risk of death. In addition, we were unable to investigate deaths by suicide or VSTED after discontinuation or rejection of MAID-PS applications. Also, any retrospective study of patient files such as this one relies on completeness and accuracy of registration, which could not be assessed.

## Conclusions

This cohort study found that the number of young patients in the Netherlands, especially females, who requested MAID-PS increased from 2012 to 2021. During this period, the majority of applications were halted by either the patient (47.3%) or the medical professional handling the application (44.8%). MAID occurred in 3.0% of cases, with suicide occurring in 4.3%. Almost all of those who died by suicide or MAID had a history of multiple psychiatric diagnoses, suicidal behavior, and intensive psychiatric treatment. Our findings point to unmet needs among female adolescents with complex psychiatric disorders and to the importance of suicide prevention when treating this group.

## Notes

Supplement. Data Sharing Statement
