# Supplementary material for: Requests for Medical Assistance in Dying by Young Dutch People With Psychiatric Disorders
Source: JAMA Psychiatry. 2025 Jan 2;82(3):246–52. doi: 10.1001/jamapsychiatry.2024.4006 (PMC11883486; doi:10.1001/jamapsychiatry.2024.4006)
Supplement: Supplement. — Data Sharing Statement [file jamapsychiatry-e244006-s001.pdf]

# Data Sharing Statement

Schweren. Requests for Medical Assistance in Dying by Young Dutch People With Psychiatric Disorder. *JAMA Psychiatry*. Published December 23, 2024.
doi:10.1001/jamapsychiatry.2024.4006

**Data**
**Data available:** Yes
**Data types:** Deidentified participant data, Data dictionary
**How to access data:** To request access, contact: l.schweren@113.nl
**When available:** With publication

**Supporting Documents**
**Document types:** None

**Additional Information**
**Who can access the data:** De-identified data regarding applications will be shared with benevolent researchers whose proposed use of the data has been approved.
**Types of analyses:** Any research purpose that directly or indirectly benefits the patient group
**Mechanisms of data availability:** with investigator support and after approval of a proposal.
**Any additional restrictions:** none
